# The Bicoid Stability Factor Controls Polyadenylation and Expression of Specific Mitochondrial mRNAs in *Drosophila melanogaster*

**DOI:** 10.1371/journal.pgen.1002324

**Published:** 2011-10-13

**Authors:** Ana Bratic, Anna Wredenberg, Sebastian Grönke, James B. Stewart, Arnaud Mourier, Benedetta Ruzzenente, Christian Kukat, Rolf Wibom, Bianca Habermann, Linda Partridge, Nils-Göran Larsson

**Affiliations:** 1 Department of Laboratory Medicine, Karolinska Institutet, Solna, Sweden; 2 Max Planck Institute for Biology of Ageing, Cologne, Germany; Stanford University School of Medicine, United States of America

## Abstract

The bicoid stability factor (BSF) of *Drosophila melanogaster* has been reported to be present in the cytoplasm, where it stabilizes the maternally contributed bicoid mRNA and binds mRNAs expressed from early zygotic genes. BSF may also have other roles, as it is ubiquitously expressed and essential for survival of adult flies. We have performed immunofluorescence and cell fractionation analyses and show here that BSF is mainly a mitochondrial protein. We studied two independent RNAi knockdown fly lines and report that reduced BSF protein levels lead to a severe respiratory deficiency and delayed development at the late larvae stage. Ubiquitous knockdown of BSF results in a severe reduction of the polyadenylation tail lengths of specific mitochondrial mRNAs, accompanied by an enrichment of unprocessed polycistronic RNA intermediates. Furthermore, we observed a significant reduction in mRNA steady state levels, despite increased *de novo* transcription. Surprisingly, mitochondrial de novo translation is increased and abnormal mitochondrial translation products are present in knockdown flies, suggesting that BSF also has a role in coordinating the mitochondrial translation in addition to its role in mRNA maturation and stability. We thus report a novel function of BSF in flies and demonstrate that it has an important intra-mitochondrial role, which is essential for maintaining mtDNA gene expression and oxidative phosphorylation.

## Author Summary

The majority of the cellular energy currency ATP is formed in a tubular network, termed mitochondria, present within virtually all eukaryotic cells. The mitochondria are unique among cellular organelles in that they contain their own genome, which encodes critical proteins necessary for cellular energy production. However, the vast majority of mitochondrial proteins are encoded in the nucleus and imported into mitochondria. Gene expression thus needs to be coordinated between the two genomes to ensure efficient mitochondrial function and sufficient adaptation to different physiological demands. The regulation of the mitochondrial genome is poorly understood, with many of the basic regulators not yet being characterized. We used RNAi in the fruit fly to study the in vivo function of the bicoid stability factor (BSF), previously thought to be a cytoplasmic and nuclear protein important for fly development. We show here that BSF is mainly localized to mitochondria, where it is essential for mtDNA gene expression, regulating the polyadenylation and maturation of specific mRNAs. Furthermore, BSF coordinates the translation and assembly of mitochondrial peptides in the inner mitochondrial membrane.

## Introduction

The maternal to zygotic transition, during which control of development shifts from maternally contributed mRNAs to genes expressed in the zygote, is of considerable interest. The maternally contributed bicoid mRNA, encoding a protein important for formation of anterior body patterning, is dependent on regulatory mechanisms controlling the cytoplasmic stability and localization of the mRNA in the zygote. The bicoid stability factor (BSF) is thought to be involved in this process as it binds the 3′UTR of the bicoid mRNA [1]. Mutation of the BSF binding site leads to reduced abundance of bicoid mRNA, whereas a P element insertion mutation that leads to a drastic reduction in BSF protein levels does not affect the abundance or distribution of endogenous bicoid mRNA [1]. In another study, BSF was reported to have a role in regulation of early zygotic genes by binding a short consensus sequence in the 5′UTR of genes expressed in the early zygote [2]. It was also reported that BSF is essential, as a P element insertion in the bsf open reading frame is lethal in the homozygous form [2]. However, homozygous bicoid mutant flies are viable and BSF therefore is likely to have an additional role besides regulating bicoid expression [2]. Characterization of global gene expression patterns in flies have shown that BSF is ubiquitously expressed in adults, further indicating that the role of BSF may not be limited to embryogenesis [3]. Studies of the subcellular localization of BSF have shown that it is present in cytoplasmic particles in oocytes and surrounding nurse cells [1] and in the cytoplasm and nucleus in early embryos [2]. Bioinformatics analyses suggest that BSF has some homology to the mammalian LRPPRC protein [1], [4], which belongs to the pentatricopeptide repeat class of proteins [5]. The LRPPRC protein has been reported to have roles in cytoplasmic RNA transport [6]–[8] and nuclear transcription [9], [10], but its main localization is in the mitochondrial matrix [4], [10], [11], where it has been suggested to stabilize mitochondrial transcripts [12].

The fly mtDNA is a small compact, circular molecule, encoding 13 essential polypeptides, which are components of the mitochondrial oxidative phosphorylation system, and RNA components of the mitochondrial translational system, which include 22 transfer RNAs and 2 ribosomal RNAs [13]. Mitochondrial transcription generates large polycistronic transcripts, which are processed by endonucleolytic cleavage to generate individual mRNAs [14], [15] These mRNAs are subsequently polyadenylated [16], in a process believed to take place in two steps. First, oligoadenylated transcripts are generated by addition of a short adenine tail to the 3‘ends of most mitochondrial mRNAs. The enzyme necessary for this oligoadenylation has not yet been identified. Second, the poly A polymerase enzyme will add up to <50 adenines to the oligoadenylated mRNAs to create the long polyadenylated tail [17].

The vast majority of the ∼10^3^ mitochondrial proteins are encoded by nuclear genes, including the majority of the respiratory chain subunits, all proteins involved in replication and transcription of mtDNA and all proteins of the mitochondrial ribosome. The regulation of oxidative phosphorylation capacity is thus dependent on a crosstalk between two genomes and nuclear genes play a key role in this process as they regulate mtDNA expression at many different levels. The mitochondrial genomes of flies and mammals have the same gene content although there are substantial differences in gene order. The high level of conservation of mtDNA and important nuclear-encoded regulators of mtDNA expression suggests that key regulatory processes are similar in insects and mammals. We therefore hypothesized that BSF might have a role in fly mitochondria, in addition to its suggested regulatory roles in developmental processes. We report here that BSF is a bona fide mitochondrial protein involved in regulating mtDNA gene expression. Contrary to previous reports, our data demonstrate that BSF is involved in the maturation and polyadenylation of mitochondrial mRNAs and coordinates mitochondrial translation. We have thus identified an essential role for BSF in maintaining mtDNA gene expression and oxidative phosphorylation in flies.

## Results

### BSF is a mitochondrial protein and the fly homologue of LRPPRC

**Figure 1:**
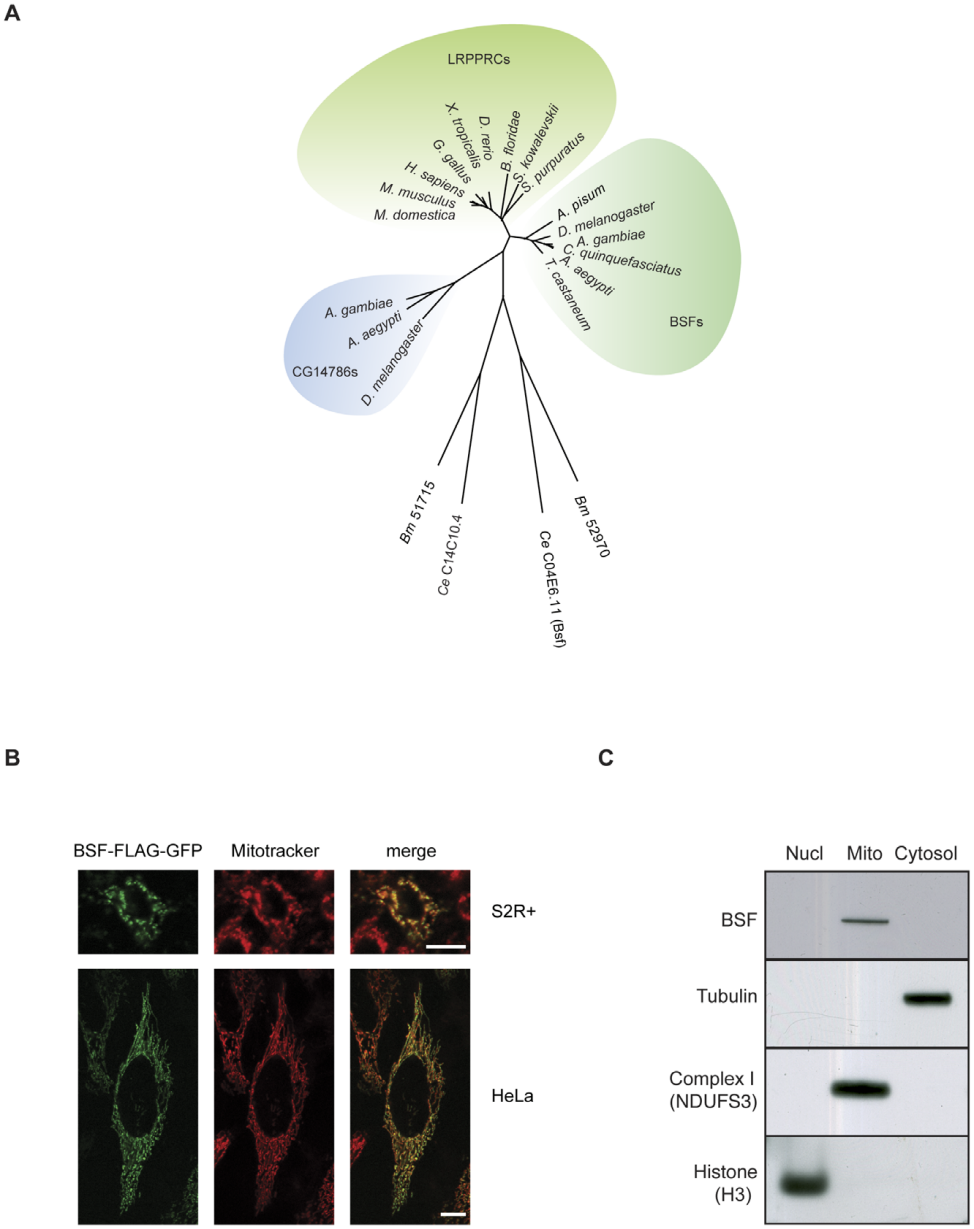
Phylogenetic analysis and subcellular localization of BSF. (A) Phylogenetic tree of the LRPPRC family of proteins. Phylogenetic analyses show two arthropod families related to LRPPRC, i.e. the Bicoid stability factor (BSF) family and the CG14786 family. The BSF family is orthologous to deuterostomian LRPPRC proteins. Dots indicate stable branches by non-parametric bootstrap analysis (found in at least 450 of 500 replicates). Orthologous sequences in nematodes are divergent, leading to long branches. Abbreviations and accession numbers are as follows: *H. sapiens*, *Homo sapiens*, NP_573566; *M. musculus*, *Mus musculus*, NP_082509; *M. domesticus*, *Monodelphis domesticus*, XP_001382190, *G. gallus*, *Gallus gallus*, XP_001234903; *X. tropicalis*, *Xenopus tropicalis*, NP_001039203; *D. rerio*, *Danio rerio*, NP_001136064; *S. kowalevskii*, *Saccoglossus kowaleskii*, XP_002734047; *S. purpuratus*, *Strongylocentrotus purpuratus*, XP_001200846; *B. floridae*, *Branchiostoma floridae*, XP_002613352; *A. pisum*, *Acyrthosiphon pisum*, BSF: XP_001944507; *D. melanogaster*, *Drosophila melanogaster*, BSF: NP_523596, CG14786: NP_569913; *A. gambiae*, *Anopheles gambiae*, BSF: XP_557938, CG14786: XP_321013; *C. quinquefasciatus*, *Culex quinquefasciatus*, BSF: XP_001846253; *A. aegypti*, *Aedes aegypti*, BSF: XP_001658446, CG14786: XP_001657384; *T. castaneum*, *Tribolium castaneum*, BSF: XP_975329. *C. elegans*, *Caenorhabditis elegans*, C04E6.11: NP_504540, C14C10.4: NP_506151; *B. malayi*, *Brugia malayi*, 52970: XP_001902062, 51715: XP_001901811. (B) S2R+ cells expressing a GFP-tagged BSF-FLAG fusion protein (BSF-FLAG-GFP), counterstained with Mitotracker Deep Red (upper panels), scale bar size 10 µm and HeLa cells expressing a GFP-tagged BSF-FLAG fusion protein (BSF-FLAG-GFP), counterstained with Mitotracker Deep Red (lower panels), scale bar size 10 µm. (C) Western blot analyses of nuclear, mitochondrial and cytoplasmic fractions to determine the subcellular localization of BSF. Antibodies against subunits NDUFS3 of mitochondrial complex I, tubulin and histone H3 were used to assess the purity of the fractions.

We previously identified two possible fly homologues for the mammalian LRPPRC protein [4]. Here we present additional phylogenetic analyses showing that BSF is the most closely related homologue to LRPPRC in flies (Figure 1A). Previous experiments have demonstrated a punctuated cytoplasmic localization of BSF in oocytes and a cytoplasmic and nuclear localization in early embryos [1], [2]. We further addressed the subcellular localization of BSF by transfecting Schneider (S2R+) and HeLa cells with a BSF-FLAG-GFP fusion construct. There was a perfect overlay between Mitotracker Deep Red and BSF-FLAG-GFP fluorescence with a co-localization rate of 91±1% in Schneider cells (N = 5) and 94±3% in HeLa cells (N = 7) (Figure 1B), thus indicating that BSF is localized to mitochondria. We also performed subcellular fractionation experiments of tissues from adult flies and found that BSF was present in the mitochondrial fraction (Figure 1C). Our results thus show that BSF is mainly localized to mitochondria, which is in good agreement with the known main localization of its mammalian homolog LRPPRC [4].

### Efficient RNAi induced knockdown of BSF in vivo

**Figure 2:**
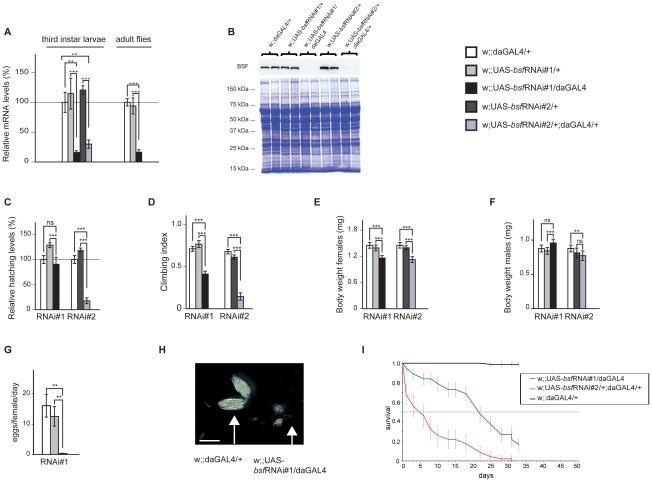
Ubiquitous bsf KD negatively affects hatching rate, climbing ability, body weight, and life span of Drosophila. (A) QRT-PCR of BSF transcript levels in third-instar control (white and grey bars) and third-instar bsf KD (black and purple bars) larvae or six-day old flies (^*^p<0.05; ^**^p<0.01; ^***^p<0.001, n = 4-5). (B) Western blot analyses were performed on mitochondrial protein preparations from third-instar larvae. Protein extracts, 10–20 µg, were separated by standard SDS-PAGE followed by Western blot analysis with antibodies against BSF. Coomassie staining of the membrane was used to assess loading. (C) The relative hatching rate in control lines (white and grey bars) and bsf KD lines (black bars). Hatching rates are shown relative to controls. ***p<0.001, Student-t-test. (D) bsf KD impairs climbing abilities of four-day old flies. ***p<0.001, Mann-Whitney U test. (E, F) Body weight of three-day-old female and male flies. ***p<0.001, Student-t–test. (G) Reduced fecundity upon ubiquitous bsf KD. **p<0.01, Student-t-test. (H) Ovaries from six-day old bsf KD females are significantly smaller (small arrow) than control ovaries (large arrow), scale bar size 1 mm. (I) bsf KD flies are short lived. Survival curves of bsf KD flies and control flies on the standard food.

In order to analyze the in vivo function of BSF, we induced gene silencing by using the UAS-GAL4 system and two independent bsf RNAi-knock down fly lines, that target different, non-overlapping regions of the bsf transcript. In each case, the performed fly crosses generated the bsf knockdown lines wDahT;+;UAS-bsf-RNAi#1/daGAL4 or wDahT;UAS-bsf-RNAi#2/+;daGAL4/+ (w;;UAS-bsfRNAi#1/daGAL4 or w;UAS-bsfRNAi#2/+;daGAL4/+) and two control lines wDahT;+;daGAL4/+ (w;;daGAL4/+), and wDahT;+;UAS-bsfRNAi#1/+;+ or wDahT;UAS-bsfRNAi#2/+;+ (w;;UAS-bsfRNAi#1/+; or w;UAS-bsfRNAi#2/+), which all were analyzed in parallel in the experiments. Ubiquitous knockdown (KD), using the daughterless-GAL4 (daGAL4) driver line, resulted in an up to 80% down-regulation of bsf transcript levels both in third-instar larvae and in adult flies (Figure 2A). Western blot analyses revealed that the BSF protein was undetectable in third-instar KD larvae (Figure 2B) and KD flies (Figure S1A), demonstrating a highly efficient KD of BSF protein expression in both bsf-RNAi lines.

### BSF deficiency affects climbing ability, fecundity, and life span in flies

Ubiquitous BSF KD caused a delay in pupal development, with the majority of pupae from line 1 hatching, and only 15% from line 2 managing to complete their eclosure (Figure 2C). KD flies had a significantly reduced climbing ability, suggesting muscle weakness (Figure 2D). Female flies from both KD lines weighed less (Figure 2E), whereas there was no consistent weight change in the males (Figure 2F). Continuous bsf KD male/female crossings resulted in no offspring, and KD flies laid significantly fewer eggs in comparison with controls (Figure 2G). Dissecting ovaries from adult bsf KD flies revealed a significant reduction in size in comparison with controls (45±21% v. 100±10%)(Figure 2H and Figure S1B), possibly contributing to the lower body weight of the female KD flies. Flies of both bsf KD lines had a drastically reduced life span compared with controls (Figure 2I), with most flies surviving less than 30 days. Together, these results identify BSF as an essential protein for fly survival.

### BSF deficiency leads to a respiratory chain dysfunction and increased lactate levels

**Figure 3:**
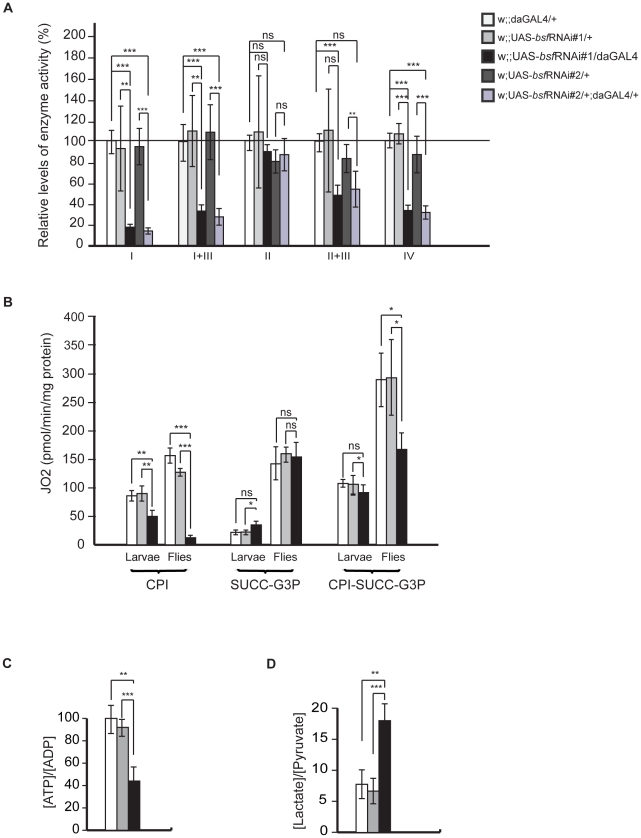
Biochemical measurements of respiratory chain function. (A) Relative enzyme activities of respiratory chain enzyme complex I (NADH coenzyme Q reductase, NQR), complex I+III (NADH cytochrome c reductase, NCR), complex II (succinate dehydrogenase, SDH), complex II+III (succinate cytochrome c reductase, SCR) and complex IV (cytochrome c oxidase, COX) in third-instar control (white and grey bars) and bsf KD (black and purple bars) larvae are shown. Bars indicate mean ± SD (^*^p<0.05; ^**^p<0.01; ^***^p<0.001; n = 6-7). (B) Oxygen consumption normalized to protein content in third-instar control (white and grey bars) and bsf KD (black bars) larvae and six-day old flies. Oxygen consumption was assessed in permeabilized larvae and thoraces from six-day old flies by using substrates entering at the level of complex I (CPI), complex II (SUCC), glycerol-3-phosphate dehydrogenase, (G3P) and complex I, II and glycerol-3-phosphate dehydrogenase (CPI-SUCC-G3P), (C) Relative ATP/ADP levels in six-day old bsf KD flies. (D) Lactate/pyruvate ratios in six-day old bsf KD flies.

To investigate the biochemical consequence of reduced BSF expression levels we measured respiratory chain enzyme activities in isolated mitochondria from larval tissue. We found that all respiratory chain complexes containing mitochondrially-encoded subunits had reduced enzyme activities. Complex I was the most affected, but complex I+III, complex II+III and complex IV activities were also profoundly reduced in bsf KD flies (Figure 3A). Complex II, containing exclusively nuclear-encoded subunits, exhibited normal enzyme activity (Figure 3A), strongly supporting the notion that loss of BSF has specific effects on mtDNA gene expression. We also analyzed mitochondrial respiratory capacity by monitoring oxygen consumption in permeabilized tissues extracted from third-instar larvae or thoraces of flies. Maximal oxygen consumption levels were significantly reduced in mitochondria from third-instar bsf KD larvae and flies in the presence of substrates entering the respiratory chain at the level of complex I (CPI), but not with substrates (SUCC-G3P) that deliver electrons to complex II or glycerol-3-phosphate dehydrogenase, which both are upstream of complex III (Figure 3B). There was significant reduction of oxygen consumption with combined substrates (CPI-SUCC-G3P) in adult fly mitochondria (Figure 3B). We also observed a progression of the decrease in the uncoupled oxygen consumption with complex I substrates, when comparing third-instar larvae and flies (Figure 3B). In summary, the results from measurements of enzyme activities (Figure 3A) and oxygen consumption (Figure 3B) show that complex I is the most affected of the oxidative phosphorylation complexes, perhaps due to its high number of mtDNA-encoded subunits. As a result of the respiratory chain deficiency the [ATP]/[ADP] ratio in adult bsf KD flies was reduced to 44% of ratio in controls (Figure 3C). Furthermore, in adult bsf KD flies the [lactate]/[pyruvate] ratio was considerably increased (Figure 3D), most likely as a consequence of the compromised respiratory function, which leads to increased lactic acid fermentation in order to maintain the reduction-oxidation homeostasis.

### BSF affects steady-state levels of mitochondrial transcripts

**Figure 4:**
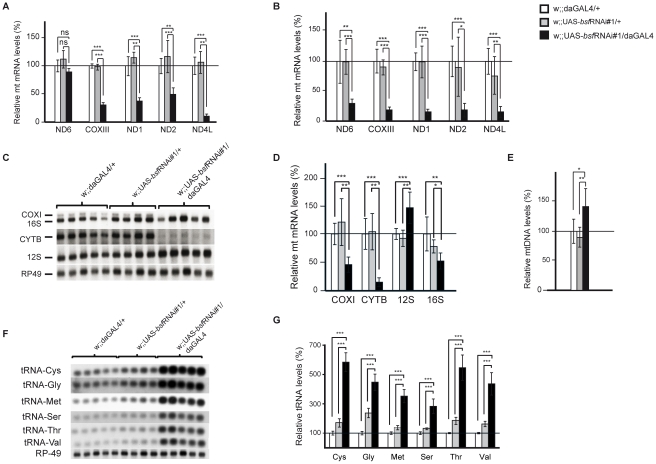
Steady-state levels of mtDNA and mitochondrial transcripts. (A-B) QRT-PCR analysis of relative levels of mitochondrial mRNAs in comparison with the nuclear ribosomal protein 49 transcript in (A) third-instar larvae and (B) six-day old flies. Northern Blot analyses (C) and quantification (D) of mitochondrial transcripts normalized to nuclear ribosomal protein 49 transcript in third-instar larvae. (E) QRT-PCR analysis of mtDNA levels in third-instar larvae. Northern blot analyses (F) and quantification (G) of mitochondrial tRNA levels in third-instar larvae. (^*^p<0.05; ^**^p<0.01; ^***^p<0.001. n = 5).

We performed a detailed study on steady-state levels of mitochondrial transcripts in bsf KD lines by using both QRT-PCR and northern blot analyses. The levels of all analyzed mitochondrial mRNAs were reduced in third-instar bsf KD larvae (Figure 4A, 4C, 4D and Figure S1C) and flies (Figure 4B), with the exception of ND6, which was significantly reduced only in adult flies. QRT-PCR demonstrated slightly increased mtDNA levels in third-instar bsf KD larvae (Figure 4E), showing that the reduced mitochondrial mRNA levels cannot be explained by mtDNA depletion. In contrast, the mitochondrially encoded small ribosomal subunit rRNA (12S rRNA) was significantly increased, while the large ribosomal subunit rRNA (16S rRNA) was slightly decreased (Figure 4C and 4D). Additionally, there was a substantial increase in levels of all analyzed mitochondrial tRNAs in third-instar bsf KD larvae (Figure 4F and 4G). There was no clear correlation between the level of a particular tRNA and the location of its gene in fly mtDNA.

### Reduced BSF level leads to increased de novo mitochondrial transcription and aberrant translation

**Figure 5:**
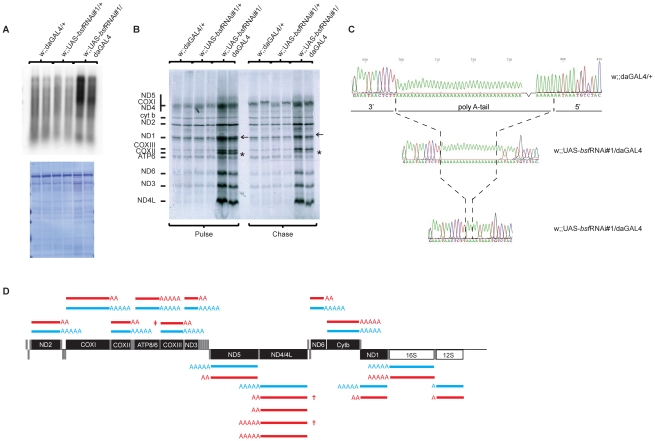
De novo mitochondrial transcription, de novo mitochondrial translation in bsf KD larvae, and polyadenylation profiles. (A) *De novo* transcription of mtDNA as determined by α^32^P-UTP incorporation. Isolated mitochondria were incubated with α^32^P-UTP and labeled transcripts were separated on MOPS/formaldehyde agarose gels and normalized to the total mitochondrial protein content per µl of sample (lower panel). (B) Analysis of de novo mitochondrial translation as determined by S^35^-methionine incorporation, normalized to the total mitochondrial protein content per µl of sample (Figure 5A, lower panel) The asterisk indicates the COXII subunit. The arrow indicates an unidentified protein migration just above the ND1 subunit. (C) Examples of electropherograms showing the polyadenylation status of the COXII mRNA in mitochondria of control (w;;daGAL4/+) and bsf KD (w;;UAS-bsfRNAi/daGAL4) larvae. (D) A summary of the polyadenylation status of mRNAs in mitochondria of third-instar controls (w;;daGAL4/+) and bsf KD (w;;UAS-bsfRNAi/daGAL4) larvae. AAAA = 40-60 adenines, AA = <20 adenines. Polycistronic transcript of COXIII-ATP6/8 (‡), antisense tRNA-Threonine (†).

The presence of increased steady-state levels of tRNAs makes it unlikely that the reduction in levels of mRNAs are explained by reduced transcription as both types of mature transcripts are produced by processing of polycistronic precursor transcripts. We nevertheless assessed mitochondrial de novo transcription by performing in organello labeling experiments. Both third-instar bsf KD larvae (Figure 5A and Figure S2B) and bsf KD flies (Figure S2A) had a dramatic increase in de novo transcription, showing that the reduced mRNA levels must be explained by increased degradation. The increased mitochondrial de novo transcription and increased mtDNA copy number are likely parts of a compensatory mitochondrial biogenesis response induced by the respiratory chain deficiency, which, in turn, is caused by defective post-transcriptional regulation of mitochondrial mRNA stability in the absence of BSF. We further investigated whether the decrease in mRNA steady-state levels resulted in decreased mitochondrial translation by assessing de novo translation in isolated mitochondria. Surprisingly, third-instar bsf KD larvae demonstrated a selective increase in the synthesis of subunits of complex I (ND1-6 and ND4L) and complex IV (COXI-III), whereas the synthesis of subunits of complex III (Cyt b) and complex V (ATP6) remained unchanged (Figure 5B). We observed an exceptionally large increase in levels of the COXII subunit of complex IV (Figure 5B, asterisk). We also found an unidentified translation product migrating above the ND1 subunit of complex I (Figure 5B, arrow). Interestingly, both of these aberrant translation products were almost invisible after a 3-hour chase with cold methionine (Figure 5B, left panel), suggesting that they are subjected to an increased degradation shortly after synthesis.

### BSF is required for polyadenylation of mitochondrial mRNAs

Decreased mitochondrial mRNA steady-state levels, in combination with increased *de novo* transcription and translation of specific mitochondrial polypeptides (ND1-6 and COX1-3) led us to investigate the nature of the mature mitochondrial mRNAs. We analyzed the 5′-and 3′-ends of mitochondrial transcripts, using RNA circularization, followed by reverse transcription and direct sequencing or cloning and subsequent sequencing (see Materials and Methods). The 5′ and 3′ ends of the 12S and 16S rRNA were identical in *bsf* KD and control larvae. However, we observed severely reduced poly A tail lengths of all mitochondrial mRNAs except Cytb and ATP6/8 (Figure 5C and 5D). Interestingly, those mRNAs with reduced poly A tail length encoded the polypeptides that showed increased levels of *de novo* synthesis. We further analyzed the 5′ and 3′ends of two polycistronic transcripts each containing an mRNA with a retained tRNA at its 3′ end (ND3 plus tRNA-Ala and COXII plus tRNA-Lys). In control samples these polycistronic transcripts were polyadenylated at their 3′-ends, showing that the polyadenylation process is not specific for fully processed mRNAs, but rather can occur at any free 3′end. In the *bsf* KD larvae, these polycistronic RNAs had severely reduced lengths of their polyA tail, thus suggesting that the lack of polyadenylation is a direct consequence of the loss of BSF and not a secondary effect of impaired translation. In the *bsf* KD larvae, we consistently failed to recover the mature COX III mRNA and only observed COXIII as part of a large polycistronic RNA. The 5′part of this RNA consisted of ATP6/8 and the 3‘part of COXIII. This RNA had undergone a correct processing at the 3′ end of COXIII but lacked polyadenylation. The lack of mature polyadenylated COXIII transcripts in the *bsf* KD larvae is thus likely explained by a combination of defective RNA processing and defective polyadenylation. Cloning and subsequent sequencing of the ND4/ND4L PCR product revealed several different RNA species in the *bsf* KD samples, indicating both polyadenylation and processing differences in comparison with control samples (Figure 5D). In summary, sequencing of RNA 5′ and 3′-ends in third-instar *bsf* KD larvae revealed a severe mRNA maturation defect with reduced poly A tail lengths and an enrichment of unprocessed polycistronic RNA intermediates containing COXIII and ND4/ND4L sequences.

### The biochemical defect in bsf KD larvae is caused by reduced levels of assembled complex I, III, and IV

**Figure 6:**
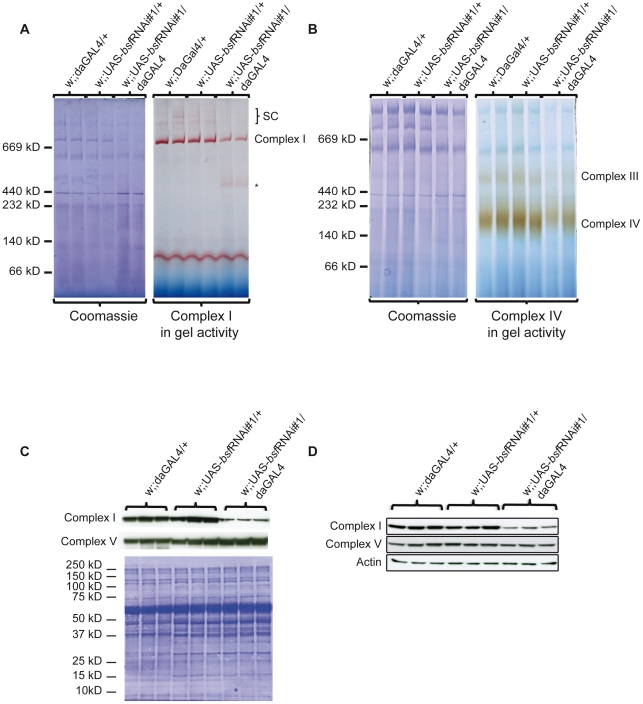
Analyses of assembled respiratory chain enzyme complexes in bsf KD larvae. (A-B) BN-PAGE analysis of mitochondrial protein extracts from third-instar bsf KD and control larvae. (A) In-gel activity of complex I, (B) In-gel activity of complex IV. (C-D) Western blot analyses of levels of nuclear encoded subunit NDUFS3 (complex I) and the α-subunit of ATP synthase (complex V) in (C) third-instar bsf KD larvae and (D) one-day-old bsf KD flies.

Despite the observed compensatory increase of de novo transcription and translation, third-instar bsf KD larvae show a severe respiratory chain dysfunction presumably causing delayed hatching and reduced lifespan. Sequencing of the mitochondrial mRNAs showed reduced length of poly A tails and processing defects, which could affect translational initiation or lead to the production of abnormal polypeptides. Subunits of complex I and IV were translated at increased rates, but a subset of the newly synthesized subunits were nevertheless preferentially degraded (Figure 5B), suggesting that they fail to assemble into mature complexes. We therefore assessed the levels of assembled respiratory chain enzyme complexes by using Blue-Native polyacrylamide gel electrophoresis (BN-PAGE). We observed reduced levels of assembled complex I, complex III, complex IV and supercomplexes in third-instar bsf KD larvae (Figure 6A and 6B) and flies (Figure S2C and S2D). The levels of assembled complex V (ATP synthase) were unaffected. This reduction in steady-state levels of assembled complexes was accompanied by reduced in-gel activity of complex I (Figure 6A, Figure S2C) and complex IV (Figure 6B, Figure S2D). The BN-PAGE and complex I in-gel activity analyses showed reduced steady-state levels of supercomplexes and the presence of a smaller, partially assembled, form of complex I in third-instar bsf KD larvae (Figure 6A, asterisk). Western blot analyses showed reduced levels of a nuclear encoded subunit of complex I (NDUFS3 subunit), indicating and supporting the notion that there is a severe reduction in steady-state levels of assembled complex I in third-instar bsf KD larvae (Figure 6C, Figure S2E) and flies (Figure 6D). There was no major reduction in steady-state levels of the nuclear-encoded complex V subunits (α-subunit of ATP synthase) in third-instar bsf KD larvae (Figure 6C, Figure S1B) and flies (Figure 6D), suggesting normal assembly. Together, these results show that third-instar bsf KD larvae and flies fail to assemble sufficient levels of complex I, III and IV as well as supercomplexes consisting of these complexes, which explains the observed profound reduction in oxidative phosphorylation capacity.

## Discussion

BSF has previously been suggested to stabilize cytoplasmic mRNAs in oocytes and early zygotic cells during the first few hours of fly embryogenesis [1], [2]. However, the ubiquitous expression of the BSF RNA [3] and the punctuate cytoplasmic localization in flies has prompted us to re-investigate the function of BSF in the fly. Surprisingly, we were able to demonstrate that the BSF protein is mainly localized to mitochondria, where it controls polyadenylation of specific mitochondrial mRNAs. Further, BSF plays a key role in the regulation of mtDNA gene expression, by coordinating mitochondrial translation in flies.

GFP-tagged BSF localized to mitochondria in transfected tissue culture cells and cell fractionation experiments showed that BSF is mainly present in mitochondria of adult flies. Interestingly, BSF was not detectable in the cytoplasmic and nuclear fractions of adult flies. It is important to note that the mammalian homolog of BSF, LRPPRC, has also been reported to have roles in regulation of cytoplasmic mRNA transport [6]-[8] and in regulation of nuclear transcription [9], [10]. However, convincing evidence proposes that the main proportion of LRPPRC is localized to mitochondria [4], [10], [11]. In one study of tissue culture cells, endogenously expressed LRPPRC was not detectable in highly purified nuclei lacking mitochondrial contamination [4]. These findings do not exclude that there is a small fraction of BSF and LRPPRC localized in other subcellular compartments besides mitochondria. However, the proposed extramitochondrial functions of BSF and LRPPRC should be revisited in a new set of experiments focusing on avoiding mitochondrial contamination of isolated nuclei and cytoplasmic extracts.

Ubiquitous RNAi-induced reduction of endogenous bsf levels in flies causes mitochondrial dysfunction with decreased respiratory chain enzyme activities and reduced levels of assembled complex I, III and IV. This mitochondrial dysfunction leads to severe phenotypes, including delayed and incomplete eclosure, reduced fecundity, sterility and shortened life span.

Mammalian cell lines with reduced LRPPRC levels have reduced mitochondrial mRNA steady-state levels and reduced mitochondrial translation [12], [18]. Interestingly, downregulation of BSF protein levels in flies also leads to decreased steady-state levels of mitochondrial mRNAs but, in contrast with the results in mammals, translation is increased and aberrant translation products are generated. Some of the mitochondrial translation products that are synthesized at increased rates in flies are subject to increased degradation, suggesting that BSF might also act as a translational coordinator. Regulation of mitochondrial gene expression in response to different metabolic demands in animal cells is largely unknown, and most likely this regulation occurs at several different levels. The basic mtDNA transcription machinery has been defined [19], but it is unclear how it is regulated. Processing of mRNAs is required for correct translation [20], but the coupling between transcript processing and translation is poorly understood. Furthermore, mitochondria are in essence a prokaryotic system with no compartmentalization between transcription and translation, which makes it likely that both of these processes directly communicate.

Here we demonstrate that the loss of BSF results in incorrect processing of the polycistronic precursor transcripts and a failure to polyadenylate a subset of both processed and polycistronic transcripts. This failure to mature mitochondrial mRNAs, in turn, leads to the destabilization of mitochondrial mRNAs and reduced steady-state levels, despite a simultaneously increased de novo transcription. In animals all mitochondrial transcripts are polyadenylated as a part of becoming a mature mRNA ready for translation, except for ND6 of vertebrates, which in humans and fish has a long 3′UTR without any poly A tail [16], [21]. In the mammalian system it is believed that nearly all mitochondrial RNAs are oligoadenylated during transcription by a yet unknown enzyme, followed by the polyadenylation to approximately 50 adenines by the mitochondrial poly A polymerase (mtPAP) [22]. A number of mitochondrial transcripts require polyadenylation to generate functional stop codons, but otherwise the exact function of mRNA polyadenylation in mitochondria is unknown. While in bacteria and chloroplasts the poly A tail seems to promote transcript degradation, eukaryotes seem to polyadenylate nearly every fully processed cytosolic mRNA at the 3′ end, resulting in increased mRNA stability, increased translational efficiency, and promotion of transport of the processed mRNA from the nucleus to the cytoplasm [23]. A primary role of BSF in the maturation of mitochondrial mRNAs is supported by the recent observation that PPR proteins in trypanosomes affect polyadenylation of mitochondrial mRNAs. In this unicellular protozoon, the PPR proteins KPAF1 and KPAF2 associate with the mtPAP, stimulating mRNA polyadenylation and thereby coordinate stability and translation of mRNA [24]. Our results suggest that BSF is involved in the actual mRNA maturation process by controlling polyadenylation of specific transcripts.

The mammalian BSF homolog LRPPRC has been shown to be stabilized by a second RNA-binding protein called SLIRP, in a direct interaction [12]. A SLIRP homolog has also been suggested to exist in flies, raising the possibility that such an interaction with a second RNA binding protein is also required in fly mitochondria. Interestingly, bioinformatics analyses identified an additional LRPPRC homolog, besides BSF, in Drosphila melanogaster [4], suggesting that both homologs work together to control the polyadenylation and translation of different sets of mitochondrial mRNAs. This is supported by our observation that several mitochondrial transcripts, such as cytb and ATP6/8, are still polyadenylated in the bsf KD lines.

Loss of BSF also results in increased and aberrant translation, and reduced levels of assembled RC complex I, III and IV, suggesting that BSF coordinates mitochondrial translation and RC complex assembly. The coupling of transcription to translation is supported by studies of the yeast homologue of BSF and LRPPRC, PET309 [25]. This factor is implicated in activation of translation by binding to the 5′UTR of yeast mitochondrial mRNAs, thereby tethering mitochondrial polysomes to the inner mitochondrial membrane, which, in turn, is thought to ensure co-translational insertion of newly synthesized polypeptides into the assembling respiratory chain complexes [26], [27]. Mitochondrial mRNAs without these 5′-UTRs are translated at normal levels followed by rapid degradation, suggesting that targeting to the inner mitochondrial membrane is necessary for stability of the newly translated peptides [28]. It is important to point out that most mRNAs encoded by metazoan mtDNA lack 5′UTRs and targeting of translation to the inner membrane of animal mitochondria, if this occurs, must therefore involve, at least partly, different regulatory mechanisms.

Cytoplasmic mRNAs have several different types of destabilizing elements and it seems that translation is coupled to degradation of some classes of mRNAs [29]. If a similar mechanism exists in mitochondria, it would suggest that the increased de novo translation in BSF KD larvae results in increased degradation of the mitochondrial mRNAs. Some support for this proposed mechanism comes from characterization of a patient with a microdeletion between the genes for ATP6/8 and COXIII, which has been reported to cause incorrect processing of the corresponding mitochondrial mRNAs and severely reduced steady-state levels of the ATP6/8 transcript due to translationally induced deadenylation [30]. Additional support comes from the observation that human cells treated with the mitochondrial translation inhibitor thiamphenicol have increased steady-state levels of mitochondrial mRNAs [31]. However, decreased translation does not always increase mRNA steady-state levels as exemplified by the conditional mouse knockout for TFB1M, which abolishes mitochondrial translation, but does not affect the levels of most mitochondrial mRNAs despite activation of de novo transcription [32] .

In conclusion, we present here the unexpected result that the BSF protein mainly is localized to mitochondria, where it controls the polyadenylation of specific mitochondrial mRNAs. In addition, our results suggests that BSF has a novel role in coordinating mitochondrial translation as loss of BSF leads to increased and uncoordinated translation with increase synthesis of unstable translation products. BSF thus has an essential role in regulating mitochondrial function in the fly.

## Materials and Methods

### Drosophila stocks and maintenance

For in vivo KD studies two independent non-overlapping UAS-bsf RNAi lines were used. w;;UAS-bsfRNAi#1 (#10302R-I) was obtained from the National Institute of Genetics (Japan) and w;UAS-bsfRNAi#2 (#22839) was obtained from the Vienna Drosophila RNAi Center (VDRC). Ubiquitous bsf knock down was achieved by crossing UAS-bsf RNAi lines to a daughterless-GAL4 (da-GAL4) driver line. UAS-RNAi lines and daGAL4 driver lines were backcrossed for at least 6 generations into the white Dahomey background (wDahT). All fly stocks were free from the endosymbiontic bacterium Wolbachia. Flies were propagated and experiments were conducted at 25°C on a 12 h∶12 h light∶dark cycle at constant humidity on a standard sugar-agar-yeast medium.

### Bioinformatics

Homologs of LRPPRC were collected using PSI-BLAST [33] against the RefSeq protein database at NCBI and aligned using ClustalX [34] with the BLOSUM matrix. Multiple sequence alignments were trimmed using the GBLOCKS server [35] with relaxed settings. The trimmed alignment was submitted to PhyML [36] using standard parameters and non-parametric bootstrap analysis with 500 replicates. The resulting tree was displayed using Dendroscope [37] and prepared for publication in Illustrator.

### Colocalization studies

Full-length bsf cDNA was obtained from the Drosophila Genomics Resource Center (SD10676, AY058795). Two amino acid changing substitutions were identified in the bsf cDNA in comparison with the reference sequence (FBtr0081087). The corresponding mutations at nucleotide positions 415 and 710 were changed by site-directed mutagenesis of the cDNA, using the QuickChange II XL Site-Directed Mutagenesis Kit (Agilent Technologies). A cDNA encoding FLAG-tagged BSF was cloned into the plasmid pAcGFP1-N2 (Clontech, Mountain View, USA) to generate the vector pbsfFLAG-AcGFP1, which encodes a fusion protein consisting of BSF-FLAG with an in-frame addition of green fluorescent protein (GFP) to its carboxy-terminus (BSF-FLAG-GFP).

Schneider 2R+ and HeLa cells were transfected in microscopy dishes (µ-Dish, ibidi, Martinsried, Germany) with pbsfFLAG-AcGFP1 using the FuGENE HD Transfection Reagent (Roche Diagnostics, Mannheim, Germany). The mitochondrial counterstaining was achieved by 100 nm MitoTracker Deep Red FM (Invitrogen, Darmstadt, Germany). Live cell image acquisition was performed with a Leica TCS SP5-X confocal microscope (Leica Microsystems, Wetzlar, Germany).

The colocalization rate was determined by the software LAS AF (Leica Microsystems, Wetzlar, Germany) under the following conditions and calculations: threshold 30%, background 20%, colocalization rate [%]  =  colocalization area/area foreground, and area foreground  =  area image − area background.

### Subcellular fractionation and Western blot analysis

All fractions were isolated from adult wDahT flies. Mitochondrial, cytoplasmic and nuclear fractions were isolated by differential centrifugation as previously described [38]. The used primary antibodies were: HISTONE H3 (Santa Cruz Biotechnology, dilution 1∶200), Complex I-subunit NDUFS3 (Mitoscience MS112, dilution 1∶1000), tubulin (Sigma, dilution 1∶1000) and polyclonal rat antisera raised against BSF (kindly provided by Professor MacDonald PM, Stanford University, dilution 1∶1000). Protein bands were visualized with ECL western blotting reagents (Bio-Rad).

Western blot analyses were performed using whole fly or mitochondrial protein extracts according to the Cell Signaling Technology protocol (CellSignaling). Additional primary antibodies used were: complex V (Mitoscience MS504, dilution 1∶5000), VDAC (Mitoscience MSAO3, dilution 1∶2000), actin (Sigma, dilution 1∶1000).

### Hatching rate, lifespan, fecundity, and climbing assays of flies

For adult hatching rate measurements eggs were collected during a 3 hours time window and transferred to vials (80 eggs/vial) to ensure standard larval density. Hatching of adult flies was monitored in regular intervals. After hatching, virgin females and males were collected and mated for 2 days.

For lifespan analyses, 50–100 females per genotype were used at a density of 10 flies per vial. Flies were transferred to new vials every two to three days and dead flies were counted. For fecundity assays, 100 females were equally distributed in ten vials, transferred to new vials every day and the number of eggs was counted.

Climbing assays were conducted and performance index was calculated as described [39]. 100 three-day-old males per genotype were tested for their climbing ability.

### DNA isolation and quantitative RT–PCR

DNA of third-instar larvae was extracted using the DNAeasy Kit (Qiagen). Mitochondrial DNA levels were determined by quantitative real-time PCR (QRT-PCR) on a 7900HT Real Time PCR system (Applied Biosystems), using SYBR green master mix (Invitrogen). Reactions were carried out in triplicates per sample in a final volume of 20 µl with 5 ng of DNA and 10 pmol of specific primers (primers are listed in Table S1).

### RNA isolation, QRT-PCR, and Northern blot analysis

Total RNA from third-instar larvae or adult flies was extracted using the Totally RNA KIT (Ambion). Reverse transcription and QRT-PCR was performed using the High capacity RNA-to-cDNA kit (Applied Biosystems) and the Taqman 2x Universal PCR mastermix, No Amperase UNG (Applied Biosystems), respectively. Custom-made TaqMan probes against Drosophila mitochondrial transcripts were obtained from Applied Biosystems and are listed in Table S1.

For Northern blot analyses, RNA was fractionated on 1.2% agarose gels and blotted to Hybond-N+ membranes (Amersham Biosciences). Membranes were hybridized with ^32^P-labeled probes and afterwards exposed to PhosphoImager Screens and/or X-ray films. Labeling of mitochondrial double-stranded DNA probes and oligonucleotides was performed as described [40]. Primers and oligonucleotides used for Northern blot are listed in Table S1.

### Biochemical evaluation of respiratory chain function

Isolation of mitochondria from third-instar larvae was performed as described [41] with modifications in buffer composition. Briefly, third-instar larvae were washed and gently homogenized in ice-cold MSB buffer (210 mM mannitol, 70 mM sucrose, 10 mM EDTA, 50 mM Tris, pH 7.5) using 15 ml Dounce homogenizers. Protein concentration was determined using Bradford assay and aliquots corresponding to 10 µg mitochondrial proteins were pelleted and resuspended in resuspension buffer (250 mM sucrose, 15 mM K2HPO4, 2 mM MgAc2, 0.5 mM EDTA and 0.5 g/L HSA, pH 7.2). Biochemical activities of respiratory chain complexes were determined as described [42].

### Respiratory rates

Third-instar larvae (n = 10) or thoraces from adult flies (n = 5) were dissected in PBS and resuspended in 2 ml of respiratory buffer (120 mM sucrose, 50 mM KCl, 20 mM Tris-HCl, 4 mM KH2PO4, 2 mM MgCl2, 1 mM EGTA, 0.01% digitonin, pH 7.2). Oxygen consumption was measured at 25°C using an oxygraph chamber (OROBOROS). Complex I-dependent respiration was assessed by adding the substrates proline (10 mM), pyruvate (10 mM), malate (5 mM) and glutamate (5 mM). Succinate and glycerol-3-phosphate dehydrogenase activities were measured using 20 mM succinate (SUCC) and 15 mM glycerol-3-phosphate (G3P), respectively. Mitochondrial quality of each sample was assessed by measuring the respiratory control rate (RCR), using 1 mM ADP (state 3) or 1 mM ADP and 2.5 µg/ml oligomycin (pseudo state 4). Permeabilized control mitochondria consistently had RCR values between 4 and 7 with complex I substrates.

The respiration was uncoupled by the addition of 400 µM CCCP and the rotenone-sensitive flux was measured in the presence of 200 µM rotenone. Finally, the protein content was determined by the Bradford method (BioRad) in order to normalize the oxygen consumption flux to mitochondrial protein content.

### ATP/ADP and lactate/pyruvate ratios

Flies (n = 10) were snap frozen directly in liquid nitrogen and kept at -80°C. Acidic extraction (PCA 7%) was performed, samples were centrifuged (16000 g, 10 min), the supernatant was neutralized with 2N KOH, 10 mM MOPS and metabolites were quantified. ADP and ATP levels were assessed as previously described [43], [44]. Briefly, ATP was quantified by using the ATPlite one step kit (PerkinElmer). For ADP levels, samples were incubated for 10 min at 37°C in 75 mM KCl, 8 mM MgSO4, 10 µg/ml pyruvate kinase and 2 mM phosphoenolpyruvate. Lactate and pyruvate concentrations were determined after 1 h incubation with horse radish peroxydase 5 U/ml, Amplex red 20 µM, 0.1 M phosphate, pH 7.2, supplemented with lactate oxidase or pyruvate oxidase, followed by fluorimetric analysis (ex:560 nm, em:590 nm), using an Infinite 200 Pro fluorimeter (Tecan).

### In organello transcription and translation assays

For the preparation of mitochondria, third-instar larvae or flies were homogenized in ice-cold isolation buffer STE+BSA (250 mM sucrose, 5 mM Tris, 2 mM EGTA, 1% (w/v) BSA, pH 7.4) using a 15 ml Dounce homogenizer. Cellular debris were pelleted at 1000 g for 5 min and supernatants were transferred to new tubes. Mitochondria were washed two times and final mitochondrial pellets were resuspend in 1 ml STE buffer in the presence of 200 µg/ml emetine (Sigma) and 100 µg/ml cycloheximide (Sigma) to inhibit cytoplasmic translation. Protein concentrations were determined using the Bradford assay.

*In organello* transcription assays were performed as described [45] using 200 µg mitochondria/sample and a modified transcription buffer (25 mM sucrose, 75 mM sorbitol, 100 mM KCl, 10 mM K_2_HPO4, 50 µM EDTA, 5 mM MgCl_2_, 1 mM ADP, 10 mM glutamate, 2.5 mM malate, 10 mM Tris-HCl (pH 7.4) and 1% (w/v) BSA). In short, after labeling, mitochondrial RNA was isolated using Totally RNA kit (Ambion). Mitochondrial RNA was fractionated on 1.2% agarose gels and blotted to Hybond-N+ membranes (Amersham Biosciences).

In vitro assays to study mitochondrial de novo translation with [^35^S]-methionine were performed as described [46]. Equal amounts of total mitochondrial protein were loaded on 15% SDS-PAGE gels. Gels were fixed in isopropanol-acetic solution, stained with Coomassie, destained in ethanol-acetic acid solution and treated with Amplify Solution (GE Healthcare). Afterwards gels were dried and [^35^S]–methionine-labelled proteins were visualized by autoradiography. The mitochondrial translation profile was compared to previously published profiles in Schneider cell lines [47], additionally ND2 and ATP6 were identified by endopeptidase fingerprinting in the second dimension (data not shown) [48].

### RNA circularization and RT–PCR

An RNA circularization protocol was modified from [49], [50]. Approximately 6 ng total mitochondrial RNA was circularized with 5 U T4 RNA ligase in 200 µl at 16°C for at least 16 h in manufacturer-supplied buffer (NEB). The circularized RNA was precipitated with an equal volume of isopropanol, incubated at -20°C for at least 4 h, and centrifuged for 20 min at top speed in a bench top centrifuge. The entire precipitate was used for complementary DNA synthesis with gene specific primers using GeneAmp RNA PCR kit (Applied Biosystems). PCR products were purified using ExoSAP-IT (Affymetrix) and sequenced. Selected PCR products were cloned into pCR-II (Invitrogen) and sequenced in order to confirm the results from direct sequencing. Primer sequences for RT-PCR and subsequent PCR are contained within Table S2.

### Blue native-polyacrylamide gel electrophoresis (BN-PAGE) and in-gel histochemistry

For BN-PAGE, 75 µg of mitochondria were pelleted and lyzed in 50 µl ice-cold digitonin buffer (1% digitonin, 20 mM Tris pH 7.4, 0.1 mM EDTA, 50 mM NaCl, 10% glycerol, 1 mM PMSF). After 15 min of incubation on ice, unsolubilized material was removed by centrifugation at 4°C. The supernatant was mixed with 5 µl of 10 x loading dye (5% (w/v) Coomassie Brilliant Blue G-250, 100 mM Tris pH 7, 500 mM 6-aminocaproic acid) and loaded on 4–10% gradient BN-PAGE gels [51], [52]. In gel complex I activity was determined by incubating the BN-PAGE gels in 2 mM Tris-HCl pH 7.4, 0.1 mg/ml NADH (Roche) and 2.5 mg/ml iodonitrozolium (Sigma). In gel complex IV activity was determined by incubating the BN-PAGE gels in 50 ml of 0.05 mM phosphate buffer pH 7.4, 25 mg 3.3′-diamidobenzidine tetrahydrochloride (DAB), 50 mg cytochrome c, 3.75 g sucrose and 1 mg catalase. All stainings were carried out at room temperature.

### Statistical analysis

Data were presented as mean ± SD. The Mann-Whitney test was used to analyze climbing index and the log-rank test was used to analyze lifespan. Unpaired t-test was used to analyze all other data statistically.

## Supporting Information

Figure S1 Phenotypic analysis and steady state level of mitochondrial transcripts in bsf KD flies. (A) Western blot analyses with antibodies against BSF were performed on mitochondrial protein preparations from six-day old bsf KD and control flies. Antibodies against actin were used to assess loading. (B) Quantification of ovary sizes in six-day old bsf KD and control flies. (C) QRT-PCR analysis of relative levels of mitochondrial mRNAs in comparison with the nuclear ribosomal protein 49 transcript in third-instar bsfRNAi#2 KD and control flies.
(TIF)

Figure S2 Level of de novo mitochondrial transcription and analyses of assembled respiratory chain complexes in bsf KD larvae and flies. (A and B) De novo transcription of mtDNA determined by α^32^P–UTP incorporation. Isolated mitochondria were incubated with α^32^P–UTP and labeled transcripts were separated on MOPS/formaldehyde agarose gels. (A) six-day old bsf KD and control flies. (B) third-instar bsf KD and control larvae. De novo transcription (left panel), and probing the same membrane to detect 16S rRNA for size comparison (right panel). (C) BN-PAGE analysis of mitochondrial protein extracts from six-day old bsf KD and control flies. The assembled respiratory chain complexes and supercomplexes are shown in the left panel. The right panel shows in-gel activity of complex I. (D) BN-PAGE analysis of mitochondrial protein extracts from six-day old bsf KD flies. The assembled respiratory chain complexes and supercomplexes are shown in the left panel. The right panel shows in-gel activity of complex IV. (E) Western blot analyses of levels of nuclear encoded subunit NDUFS3 (complex I) and the α-subunit of ATP synthase (complex V) in third-instar bsf KD larvae. Antibodies against VDAC were used to assess loading.
(TIF)

Table S1 List of oligonucleotide sequences and Taqman probes used for cloning of the BSF-FLAG-GFP construct and quantification of steady-state levels of mtDNA, mt-tRNAs and mt-mRNAs.
(TIF)

Table S2 List of oligonucleotide sequences used for RT PCR and subsequent PCR to determine the polyadenylation profile of mt transcripts. RT-PCR was done using the forward primer (F) and subsequent PCR for sequencing was done using the forward primer in combination with the reverse (R) primer. Forward primer for COXI and additional primer sequences used for polyadenylation sequencing were designed according to Stewart and Beckenbach, 2009 [50].
(TIF)
