# Supplementary figures and images for: The Bicoid Stability Factor Controls Polyadenylation and Expression of Specific Mitochondrial mRNAs in Drosophila melanogaster
Source: PLoS Genet. 2011 Oct 13;7(10):e1002324. doi: 10.1371/journal.pgen.1002324 (PMC3192837; doi:10.1371/journal.pgen.1002324)

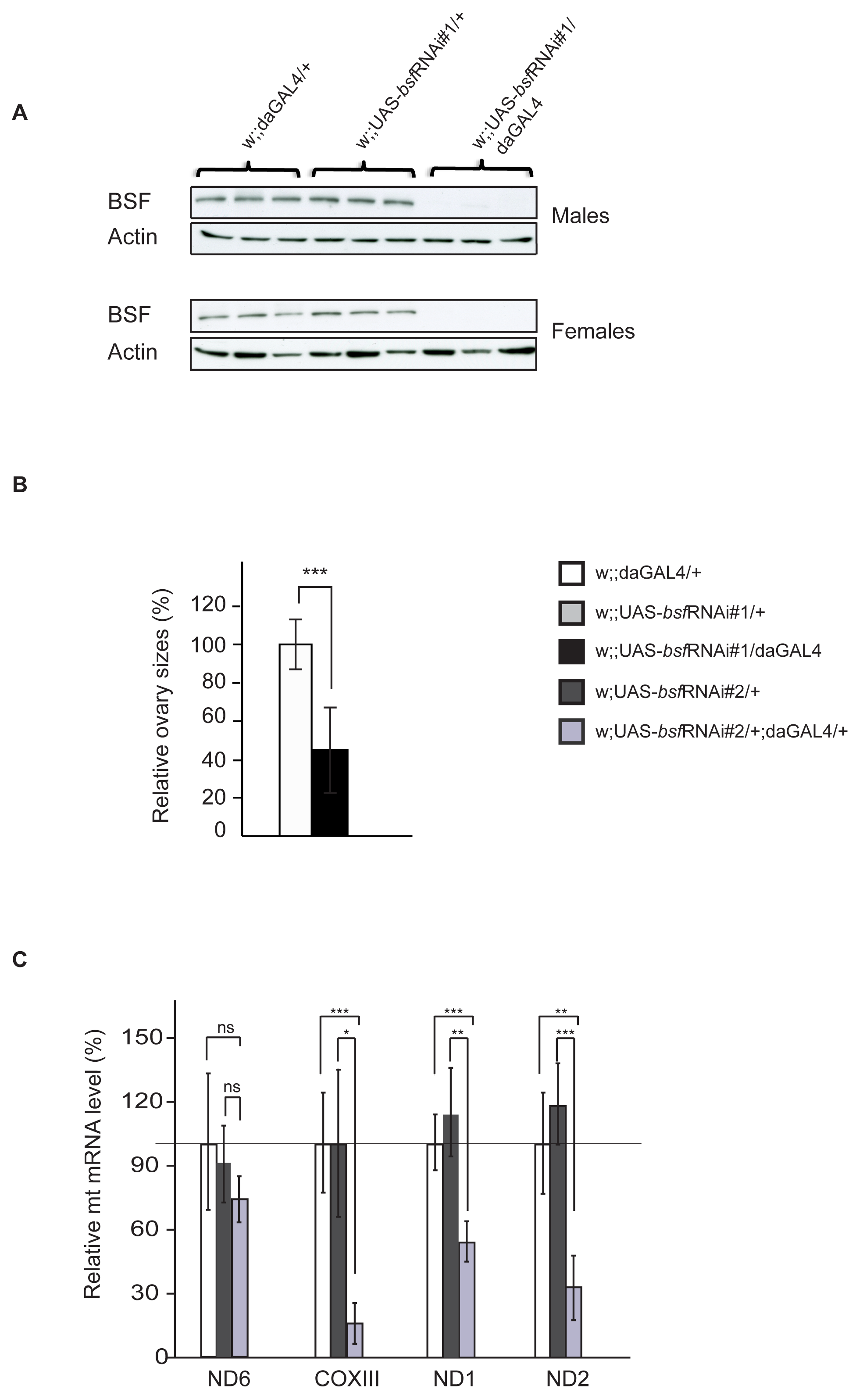

Supplement: Figure S1 — Phenotypic analysis and steady state level of mitochondrial transcripts in bsf KD flies. (A) Western blot analyses with antibodies against BSF were performed on mitochondrial protein preparations from six-day old bsf KD and control flies. Antibodies against actin were used to assess loading. (B) Quantification of ovary sizes in six-day old bsf KD and control flies. (C) QRT-PCR analysis of relative levels of mitochondrial mRNAs in comparison with the nuclear ribosomal protein 49 transcript in third-instar bsfRNAi#2 KD and control flies. (TIF) [file pgen.1002324.s001.tif]

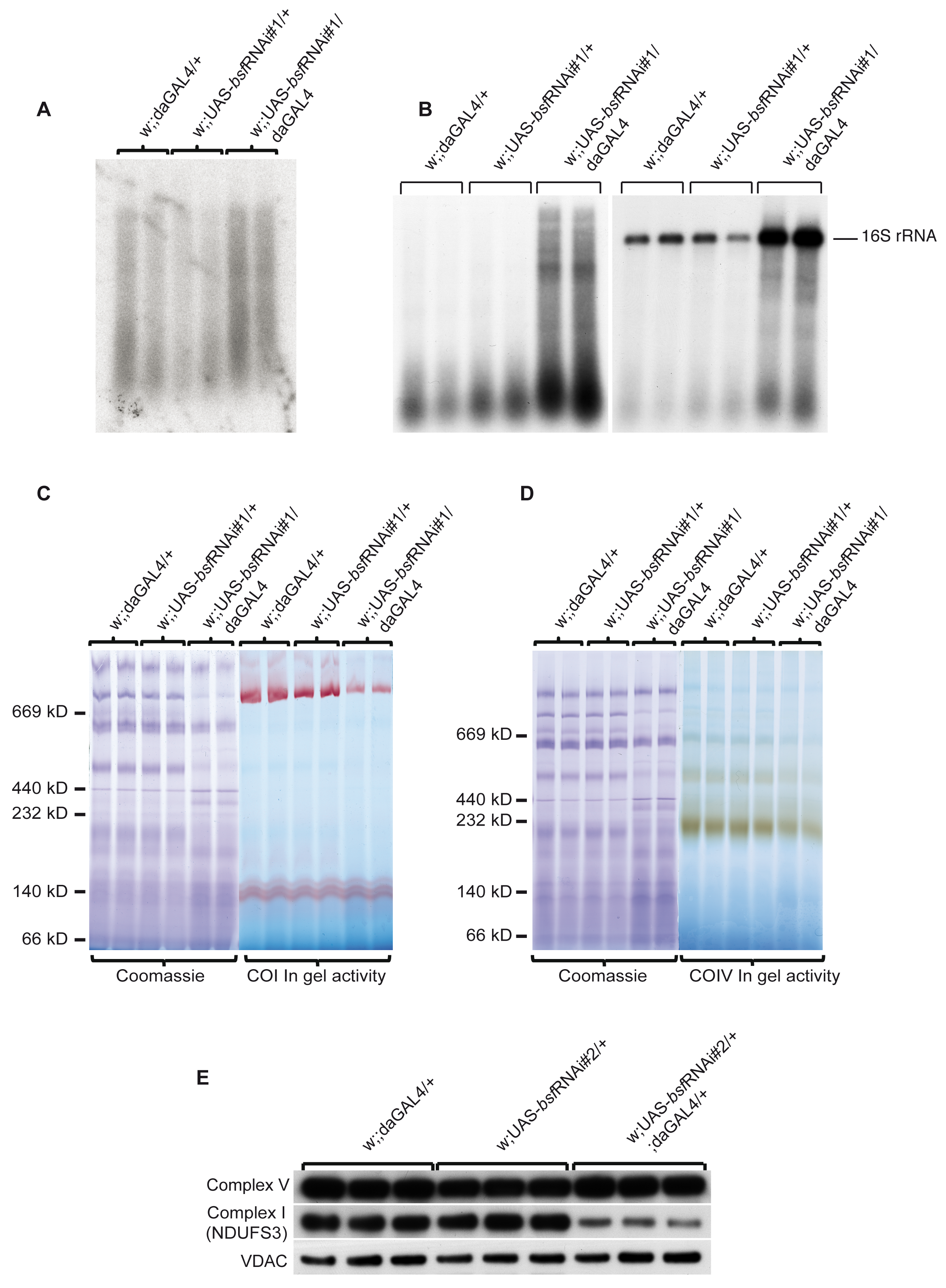

Supplement: Figure S2 — Level of de novo mitochondrial transcription and analyses of assembled respiratory chain complexes in bsf KD larvae and flies. (A and B) De novo transcription of mtDNA determined by α32P–UTP incorporation. Isolated mitochondria were incubated with α32P–UTP and labeled transcripts were separated on MOPS/formaldehyde agarose gels. (A) six-day old bsf KD and control flies. (B) third-instar bsf KD and control larvae. De novo transcription (left panel), and probing the same membrane to detect 16S rRNA for size comparison (right panel). (C) BN-PAGE analysis of mitochondrial protein extracts from six-day old bsf KD and control flies. The assembled respiratory chain complexes and supercomplexes are shown in the left panel. The right panel shows in-gel activity of complex I. (D) BN-PAGE analysis of mitochondrial protein extracts from six-day old bsf KD flies. The assembled respiratory chain complexes and supercomplexes are shown in the left panel. The right panel shows in-gel activity of complex IV. (E) Western blot analyses of levels of nuclear encoded subunit NDUFS3 (complex I) and the α-subunit of ATP synthase (complex V) in third-instar bsf KD larvae. Antibodies against VDAC were used to assess loading. (TIF) [file pgen.1002324.s002.tif]

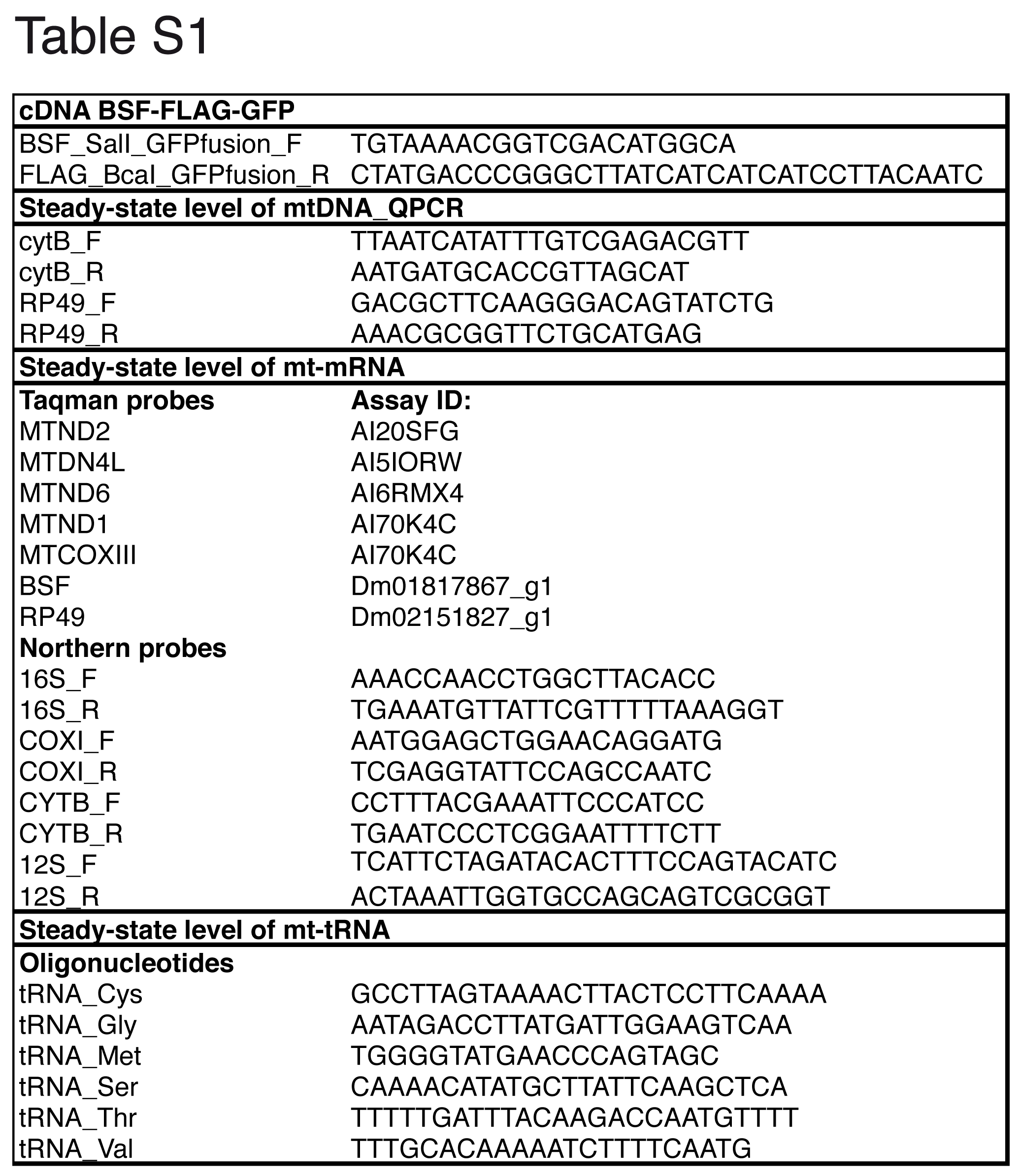

Supplement: Table S1 — List of oligonucleotide sequences and Taqman probes used for cloning of the BSF-FLAG-GFP construct and quantification of steady-state levels of mtDNA, mt-tRNAs and mt-mRNAs. (TIF) [file pgen.1002324.s003.tif]

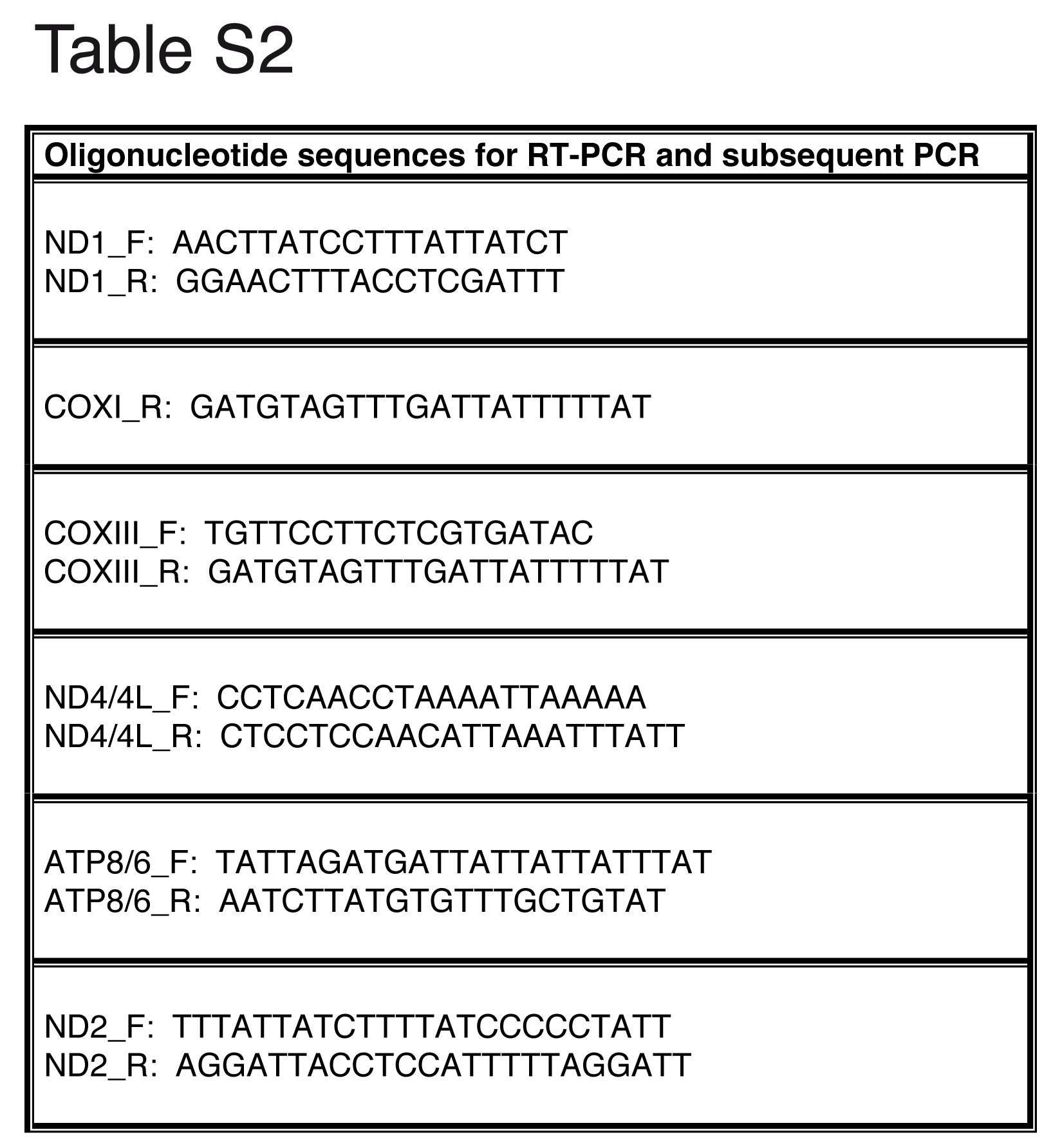

Supplement: Table S2 — List of oligonucleotide sequences used for RT PCR and subsequent PCR to determine the polyadenylation profile of mt transcripts. RT-PCR was done using the forward primer (F) and subsequent PCR for sequencing was done using the forward primer in combination with the reverse (R) primer. Forward primer for COXI and additional primer sequences used for polyadenylation sequencing were designed according to Stewart and Beckenbach, 2009 [50]. (TIF) [file pgen.1002324.s004.tif]
